# Catalyzing change: a literature review on the implementation of the Nature Futures Framework

**DOI:** 10.1007/s11625-025-01682-y

**Published:** 2025-06-27

**Authors:** Sana Okayasu, Jan J. Kuiper, Ghassan Halouani, HyeJin Kim, Brian W. Miller, América Paz Durán, Angelique Vermeer, Machteld Schoolenberg, Shizuka Hashimoto, Carolyn Lundquist

**Affiliations:** 1 PBL Netherlands Environmental Assessment Agency, The Hague, The Netherlands; 2 Stockholm Resilience Centre, Stockholm University, Stockholm, Sweden; 3 Ifremer, HMMN, Laboratoire Ressources Halieutiques, 62200 Boulogne-sur-Mer, France; 4 UK Centre for Ecology and Hydrology, Lancaster, UK; 5 U.S. Geological Survey, North Central Climate Adaptation Science Center, Boulder, CO USA; 6 Instituto de Ecología y Biodiversidad (IEB-Chile), Santiago, Chile; 7 The University of Tokyo, Tokyo, Japan; 8 School of Environment, University of Auckland, Auckland, New Zealand; 9 National Institute of Water and Atmospheric Research, Hamilton, New Zealand

**Keywords:** IPBES, Scenarios, Models, Visions, Value plurality, Transformative change

## Abstract

The Nature Futures Framework (NFF), developed under the Intergovernmental Science–Policy Platform on Biodiversity and Ecosystem Services (IPBES), serves as a catalyst for advancing new scenarios and models focused on biodiversity and ecosystem services within the broader research community. In particular, the framework facilitates the development of scenarios and models that can help guide change processes toward desirable futures for nature and people. This paper assesses 31 studies that have engaged with the NFF since its introduction in 2020, aiming to identify which research areas have been addressed, and where development needs remain. The applications exhibit a large diversity in terms of locations, spatial scales, methods, outputs, and stakeholder involvement. The most common use of the framework has been in developing visions and scenarios. Nearly all studies engaged with diverse values of nature through the framework’s fundamental value perspectives: ‘*Nature for Society*’, ‘*Nature for Natur*e’, and ‘*Nature as Culture*/*One with Nature*’. While the framework is generally perceived as useful, challenges remain in integrating the NFF across multiple scales and fully incorporating plural values, particularly in measuring relational aspects and avoiding Western-centric biases. Future research priorities include developing integrated, quantitative studies and exploring transformative pathways to enhance the framework's effectiveness in driving sustainable outcomes. Overall, the growing body of work using the NFF provides a strong foundation for distilling best practices, facilitating large-scale applications, and achieving the framework's objectives.

**Supplementary Information:**

The online version contains supplementary material available at https://doi.org/10.1007/s11625-025-01682-y.

## Introduction

Scenarios and models are indispensable tools for conducting integrated environmental assessments, especially when it comes to evaluating the future outcomes of the actions we take today (Van Vuuren et al. 2012). Recognizing their pivotal role, one of the first assessments conducted by the Intergovernmental Science–Policy Platform on Biodiversity and Ecosystem Services (IPBES) was a methodological assessment on scenarios and models of biodiversity and ecosystem services (IPBES 2016). The assessment shed light on several limitations of existing scenario approaches in relation to the needs of IPBES. These include the scarcity of scenarios and models that put nature at the center; describe futures that are positive for nature; operationalize transformative change; are multi-scale; engage with people’s values and value pluralism; and are co-produced through inclusive, participatory processes (IPBES 2016; Kok et al. 2016; Rosa et al. 2017; Pereira et al. 2020).

Responding to these conclusions of the Methodological Assessment, the IPBES Plenary mandated an expert group (2016–2019) and task force (2019–2023) on scenarios and models to catalyze the development of new scenarios and models that can better inform policymaking for nature and nature’s contributions to people (annex II to decision IPBES-7/1). The expert group set out a transdisciplinary research strategy to address the shortcomings presented above (Rosa et al. 2017), culminating in the presentation of the Nature Futures Framework (NFF), a flexible tool to support the development of scenarios and models of desirable futures for people, nature and Mother Earth (IPBES 2023). At the 9th IPBES Plenary in 2022, the member states welcomed the progress made by the task force on scenarios and models, including on the NFF, and invited the scientific community and other relevant actors, in particular Indigenous peoples and local communities, to discuss the opportunities and limits of, as well as test, as appropriate, the NFF (Decision IPBES-9/1).

The NFF positions three value perspectives that people use to relate to nature—*Nature for Nature*; *Nature as Culture*/*One with Nature*; and *Nature for Society—*at the vertices of a triangle, providing reference points that are applicable across spatiotemporal scales and regions, offering a simple structure for consistency in the scenarios and models that use it. At the same time, they open an interior space for exploring the plurality of desirable people–nature relationships in resonance with local realities, based on which an infinite number of scenarios can be co-created (Pereira et al. 2020). The framework thus distinguishes itself by focusing on desirable futures for people and nature, and the transformative changes needed to achieve them. As such, especially when quantified with models, nature futures scenarios may be used to substantiate and develop pathways toward the internationally agreed 2050 Vision “Living in harmony with nature” of the Convention on Biological Diversity (CBD) and related internationally agreed environmental policy targets. In 2023, the IPBES task force on scenarios and models released a rolling draft of the methodological guidance of the NFF to illustrate possible uses of this framework and to catalyze its application among research communities and knowledge holders (IPBES 2023).

The NFF and its accompanying methodological guidance are not intended to be prescriptive, or to provide highly specific, predefined characteristics for scenarios. Instead, the framework is presented as a ‘flexible tool’ that can be integrated with various scenario methods and adopted to a variety of contexts and scales, combining an inductive and deductive approach to scenario development (Pereira et al. 2021b). This reflects the complexity involved in describing nature futures across different places, as well as the imperative for collaboratively produced, multi-scale scenarios. It also reflects the mandate from IPBES underlying the NFF, which is to catalyze, not to create. It is up to the broader scientific community and other relevant actors to leverage the NFF for developing new scenario and model applications. Correspondingly, in the work plan of the next task force on scenarios and models, as decided at IPBES-10, there is a strong emphasis on engaging with communities of practice to further drive the catalyzation of the development of new scenarios and models. An important implication is that, to determine whether or not the NFF is able to fulfill its promise, studies using the NFF need to be assessed and synthesized. As the saying goes, the proof of the pudding is in the eating.

Here, we present the outcomes of a review of the literature that has actively engaged with the NFF since its presentation to the literature in 2020 (Pereira et al. 2020), revealing emerging patterns of application. We discuss these findings in relation to the key knowledge gaps that the NFF addresses. The purpose of this review is to offer insights into the adoption and practical application of the NFF, while identifying remaining knowledge gaps to inspire and guide future use.

## Methods

### Search strategy

For this study, we focused on peer-reviewed scientific literature. On January 12th 2024, we conducted a systematic search of literature in ‘Scopus’ to identify all published studies that used the NFF. The following search strings were used to identify articles that mentioned the NFF: *ALL ("Nature Futures Framework" OR "Natures Futures Framework" OR "Nature Future Framework"),* and *ALL ("Nature" AND {Futures Framework})*. The literature identified through the systematic search as using or explicitly mentioning the NFF was uploaded to a library on Zotero.org, available at: https://www.zotero.org/groups/4937409/nature_futures_framework/library. We discuss the limitations of our search strategy, such as the omission of gray literature, in “Discussion”.

### Data extraction and analysis

For the purpose of this review, we screened all resulting articles and excluded the articles that merely mentioned or cited the NFF, to identify those articles that actively engaged with the framework. All selected studies were collected in a datasheet. From each study, we extracted diverse information to help better understand the main uses of the NFF, the produced outcomes, and where gaps in applications persist (the entire set of variables and codes can be found in Table S1).

For each study, we collected basic information like the year of publication, authors, title, source, and location of the (case) study. To further characterize the studies, we adopted the six categories used by Biosphere Futures, an online database of scenario case studies (Kuiper et al. 2024), to describe the main uses of the NFF: (i) as a tool for *creating new visions and scenarios*; (ii) as a tool to *discuss the NFF* in relation to other/new concepts, theories and methods; (iii) as a *classification tool* (using the NFF perspectives as a typology for classification and assessment of existing information); (iv) as a tool for *interpreting and translating existing scenarios*; (v) as a tool for *adapting and developing quantitative models*; and (vi) as an *exploration tool* (applying the NFF to a system or problem setting to ‘open up’ for more plural perspectives on nature and identify diverse values, associated indicators, and/or relevant data for monitoring). We also applied several other categories used by Biosphere Futures to code scenario case studies. These categories, each with a set of predefined options, included: *Spatial scale; Main ecosystem type; Methods used; Stakeholder engagement; Produced results* (see Table S1 for all sub-categories).

We added some categories to collect more information specific to the NFF and how its application is associated with existing frameworks and global initiatives. For each study, we coded which of the NFF perspectives (*Nature for Society; Nature for Nature; Nature as Culture/One with Nature*) were addressed, and how they related to the scenario typology from the IPBES assessment on scenarios and models (*Exploratory scenarios; Target-seeking scenarios; Policy screening scenarios; Retrospective policy evaluation*), as well as the policy cycle as employed by IPBES (*Agenda setting, Policy design, Policy implementation, and Policy review*). We also coded whether the studies mentioned the Global Biodiversity Framework (GBF) of the Convention on Biological Diversity (either as the 2050 Vision “Living in harmony with nature”, Aichi-targets, post-2020 GBF, or the Kunming–Montreal GBF); the Sustainable Development Goals (SDGs) of the UN Agenda 2030; the concept of nature’s contributions to people (NCPs); or the concept of transformative change. Finally, we extracted qualitative expressions from the studies on the strengths and limitations of using the NFF.

We first report on the results of the systematic search. We then present descriptive statistics and qualitative assessment of the information we collected from the studies that engaged with the NFF. This provides a first comprehensive overview of papers that have engaged with the NFF, revealing emerging patterns of application. These insights enable us to discuss these patterns in relation to the key knowledge gaps that the NFF addresses to identify remaining research needs, while already serving to stimulate future uptake by showcasing the diverse array of use cases.

## Results

### Systematic search

The systematic literature search identified 75 scientific publications which either use or mention the NFF. Of these publications, 31 provide in-depth application or discussion of the NFF and are further analyzed below. The 44 remaining articles refer to the NFF but without substantial discussion, and therefore were not considered in the descriptive analysis. Table 1 provides a summarized overview of the 31 publications analyzed. The full details can be found in Table S1.

**Table 1:** Overview of NFF applications clustered per type of use

Type of NFF use	Use of quantitative methods	Stakeholder engagement	Methods/approach used	Types of results produced	Publication ID
Developing visions and scenarios (12 studies)	No (10)	Yes (8)	- Workshops and deliberation (7) - Mapping (7) - Desktop study (4) - Surveys and interviews (2) - Art-based practices (2) - Games (1)	- Scientific publication(s) (8) - Drawing(s)/diagram(s) (6) - Narrative(s) (5) - Booklet/other publication(s) (2) - Collage(s) (2) - Storyline(s) (1) - Oral presentation(s) (1) - Map(s) (1)	Yu et al. (2021) Durán et al. (2023) Rana et al. (2020) Mansur et al. (2022) Mayer et al. (2023) Pereira et al. (2023) Kuiper et al. (2022) Lembi et al. (2020)
		No (2)	- Desktop study (2) - Mapping (2)	- Scientific publication(s) (2) - Drawing(s)/diagram(s) (2) - Narrative(s) (1)	Dasgupta and Shakya (2023) Sarkki et al. (2023)
	Yes (2)	No (2)	- Modeling (2) - Desktop study (2) - Mapping (2)	- Scientific publication(s) (2) - Quantitative analyses (2) - Quantitative changes in key variables in data set(s) (2) - Drawing(s)/diagram(s) (2) - Narrative(s) (1) - Map(s) (1)	Haga et al. (2023) Dou et al. (2023)
Discussing the NFF (8 studies)	No (7)	Yes (5)	- Workshops and deliberation (5) - Desktop study (3) - Mapping (3) - Art-based practices (1)	- Scientific publication(s) (5) - Narrative(s) (2) - Drawing(s)/diagram(s) (3) - Technical report(s) (2) - Theater(s) (1) - Collage(s) (1)	Yu et al. (2021) Mansur et al. (2022) Pereira et al. (2020) Kim et al. (2023) Rosa et al. (2020a)
		No (2)	- Desktop study (2)	- Scientific publication(s) (1)	Kluiving and Waterman (2023) Greenway (2022)
	Yes (1)	No (1)	- Desktop study (1)	- Scientific publication(s) (1)	Kramer et al. (2023)
Classification tool (7 studies)	No (6)	Yes (3)	- Surveys and interviews (3) - Mapping (2) - Workshops and deliberation (1)	- Drawing(s)/diagram(s) (3) - Scientific publication(s) (3) - Quantitative analyses (2)	Diprose et al. (2022) Dunn-Capper et al. (2023) Stronge et al. (2023)
		No (3)	- Desktop study (3) - Mapping (3) - Surveys and interviews (1)	- Scientific publication(s) (3) - Drawing(s)/diagram(s) (3)	de Bruin et al. (2023) Lahoti et al. (2023) Shaikh & Hamel (2023)
	Yes (1)	No (1)	- Modeling (1) - Desktop study (1)	- Scientific publication(s) (1) - Quantitative analyses (1) - Quantitative changes in key variables in data set(s) (1) - Map(s) (1)	Henry et al. (2022)
Translating scenarios (5 studies)	No (3)	Yes (1)	- Workshops and deliberation (1) - Desktop study (1) - Mapping (1)	- Scientific publication(s) (1) - Narrative(s) (1) - Drawing(s)/diagram(s) (1) - Booklet/other publication(s) (1) - Collage(s) (1)	Durán et al. (2023)
		No (2)	- Desktop study (2) - Mapping (2) - Surveys and interviews (1)	- Scientific publication(s) (2) - Drawing(s)/diagram(s) (2)	Quintero-Uribe et al. (2022) de Bruin et al. (2023)
	Yes (2)	No (2)	- Modeling (2) - Desktop study (2) - Mapping (2)	- Scientific publication(s) (2) - Drawing(s)/diagram(s) (2) - Quantitative analyses (2) - Quantitative changes in key variables in data set(s) (1) - Narrative(s) (1)	Haga et al. (2023) Alexander et al. (2023)
Adapting and developing models (4 studies)	No (1)	Yes (1)	- Workshops and deliberation (1) - Desktop study (1) - Mapping (1)	- Scientific publication(s) (1) - Drawing(s)/diagram(s) (1) - Technical report(s) (1)	Kim et al. (2023)
	Yes (3)	No (3)	- Modeling (2) - Desktop study (2) - Mapping (1)	- Scientific publication(s) (3) - Quantitative analyses (2) - Quantitative changes in key variables in data set(s) (2) - Narrative(s) (1) - Drawing(s)/diagram(s) (1) - Map(s) (1)	Kramer et al. (2023) Haga et al. (2023) Wen et al. (2022)
Exploration tool (3 studies)	No (3)	Yes (3)	- Workshops and deliberation (3) - Desktop study (2) - Mapping (2)	- Scientific publication(s) (3) - Drawing(s)/diagram(s) (2) - Narrative(s) (1)	Palacios-Abrantes et al. (2022) Resende et al. (2020) Sarkar et al. (2020)

Results show a variety of both qualitative and quantitative applications, combining diverse methods with engagement of stakeholders or expert-based analysis. Some NFF applications have combined use types, thus appearing multiple times in the table

### Analysis and synthesis

#### Publication year, source, and authorship

The paper from the IPBES expert group on scenarios and models that introduced the NFF to the scientific literature (Pereira et al. 2020) was published in 2020. In the same year, a set of papers was published that were linked to a youth workshop and summer school that were co-organized by the IPBES expert group on scenarios and models. After that, it took some time for studies using the NFF to take place and for results to appear in the literature, with only one source using the NFF in 2021. However, the two years thereafter have provided a clear sign of adoption, with nine articles published in 2022 and 15 articles in 2023. These articles were published in a broad range of academic journals in the environmental sciences (16 different titles). *Sustainability Science* (10 articles) stands out, which can be explained by a special issue on the NFF (this issue). An examination of authorship across the 31 publications identified four papers led by co-authors of Pereira et al. (2020) (Rosa et al. 2020a; Kuiper et al. 2022; Kim et al. 2023; Pereira et al. 2023), and nine other papers co-authored by an overlapping group of authors. This is evidence of an active effort from members of the former IPBES expert group on scenarios and models to disseminate the NFF. In contrast, 17 papers were published by authors not engaged in the initial publication by Pereira et al. (2020), indicating an expansion of the user group of the NFF.

#### Application of the NFF

**Fig. 1:**
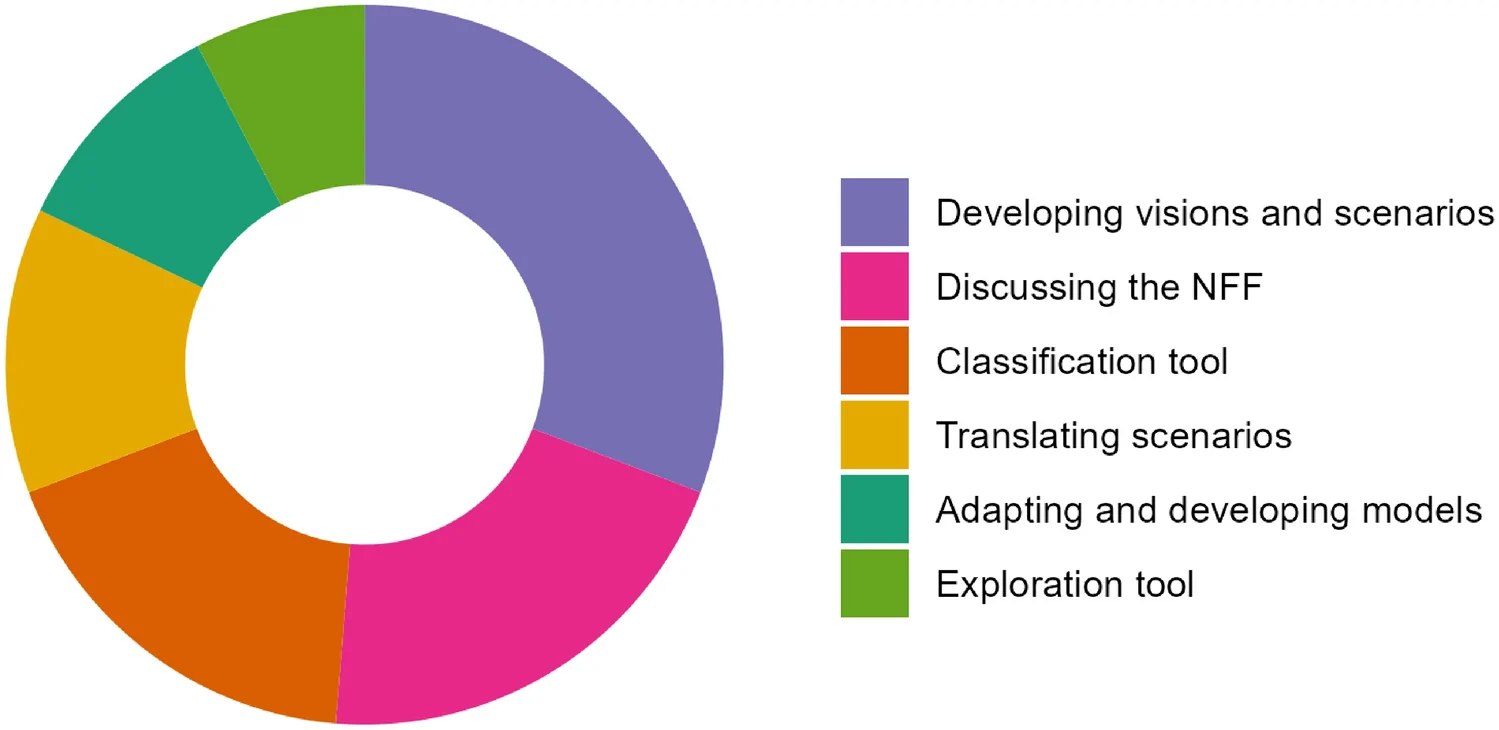
The proportions of predefined use categories of the NFF. Some NFF applications have combined multiple use types, making the total count (*n* = 39) higher than the number of studies (31 studies)

The NFF has been used for a variety of purposes, covering all the predefined use categories (Fig. 1; Table S1). About a third of the 31 studies are examples of use of the NFF for its primary purpose, that is, creating visions and scenarios (*n* = 12) (Box 1–2). The second most common purpose was the use of the NFF and its value perspectives to discuss relevant concepts, theories, and methods (*n* = 8) (Box 3). Other studies in our dataset used the NFF as a classification tool (*n* = 7) (Box 4), as a tool for translating existing scenarios (*n* = 5) (Box 5), for adapting and developing models (n = 4) (Box 6), or to provide guidance in exploring and identifying various information on people–nature relationships (*n* = 3) (Box 7). These different uses were not mutually exclusive and were combined in some of the studies (*n* = 7).

Box 1: Developing visions and scenarios—Example of application of the NFF in developing visions of transformed human–nature relationships on the high seas (Pereira et al. 2023) This study organized a futures thinking process that uses the NFF as a mechanism to transform how humans conceptualize the high seas and thereby the governance of the ocean. Through a series of workshops with high-seas experts that combined the NFF, participatory scenario-building methods, and artistic creation, this study defined transformative visions and pathways for the high seas in line with the three value perspectives of the NFF.

**Figure:**
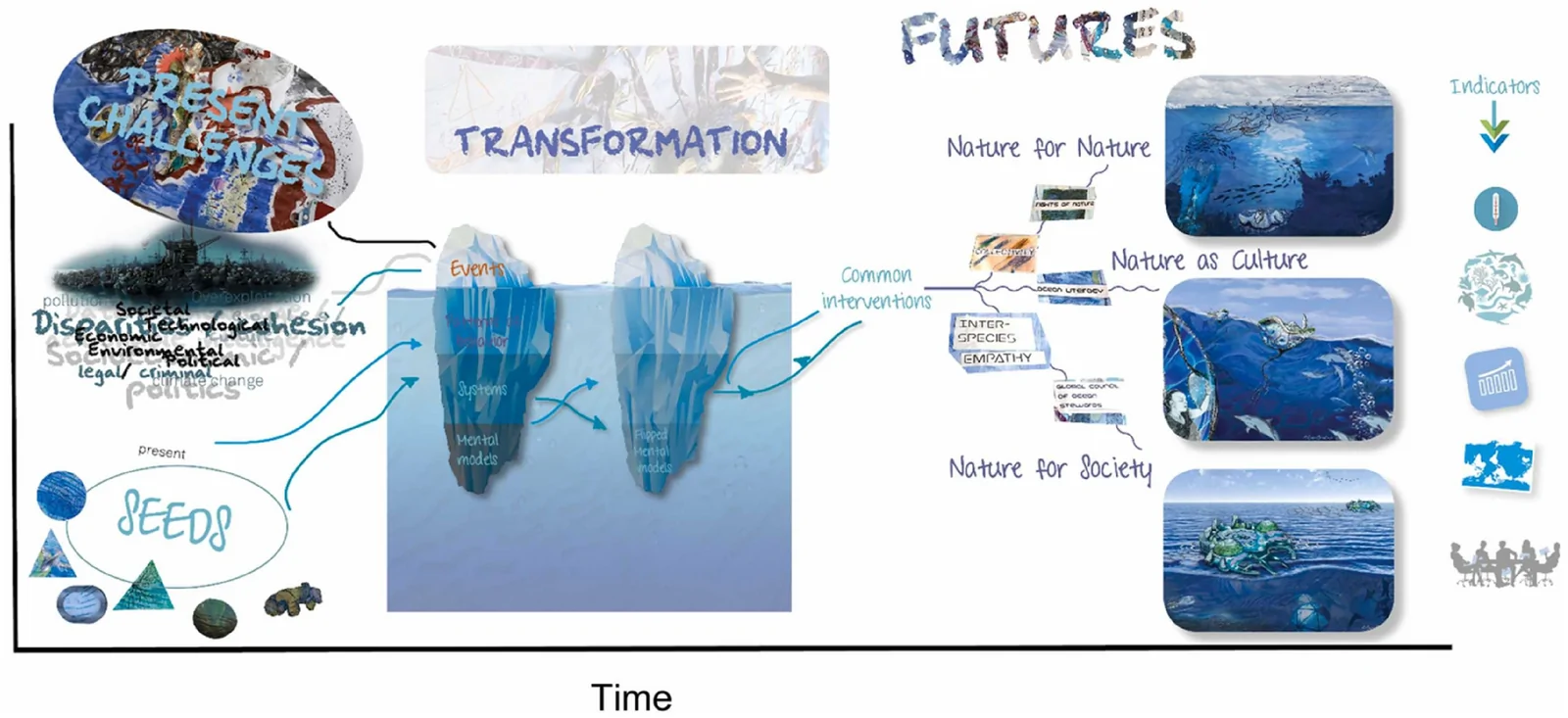
Overarching process adapting the three horizons framework. Starting with challenges and seed ideas in the present (left side) and then moving through a transformative process of flipping paradigms through the iceberg model (middle), which offers the common starting points for the three more preferable futures to emerge on the right and brainstorming the indicators that would be needed to measure progress. Source: Pereira et al. (2023)

Box 2: Developing visions and scenarios – Example of application of the NFF in simulating desirable land use scenarios in Europe in 2050 (Dou et al. 2023) This study used the NFF as a lens for developing plural land use scenarios for spatially explicit modeling in Europe for 2050. The authors developed three positive scenarios each representing a value perspective of the NFF: favoring land systems providing strong climate regulation (nature for society), species conservation (nature for nature), or agricultural heritage features (nature as culture). A spatially explicit model (CLUMondo) was then used to simulate how these scenarios would play out across the European land systems in 2050. Results showed a need for drastic change in European land systems for all three desirable futures and identified areas of synergies and trade-offs depending on which value perspectives are prioritized

**Figure:**
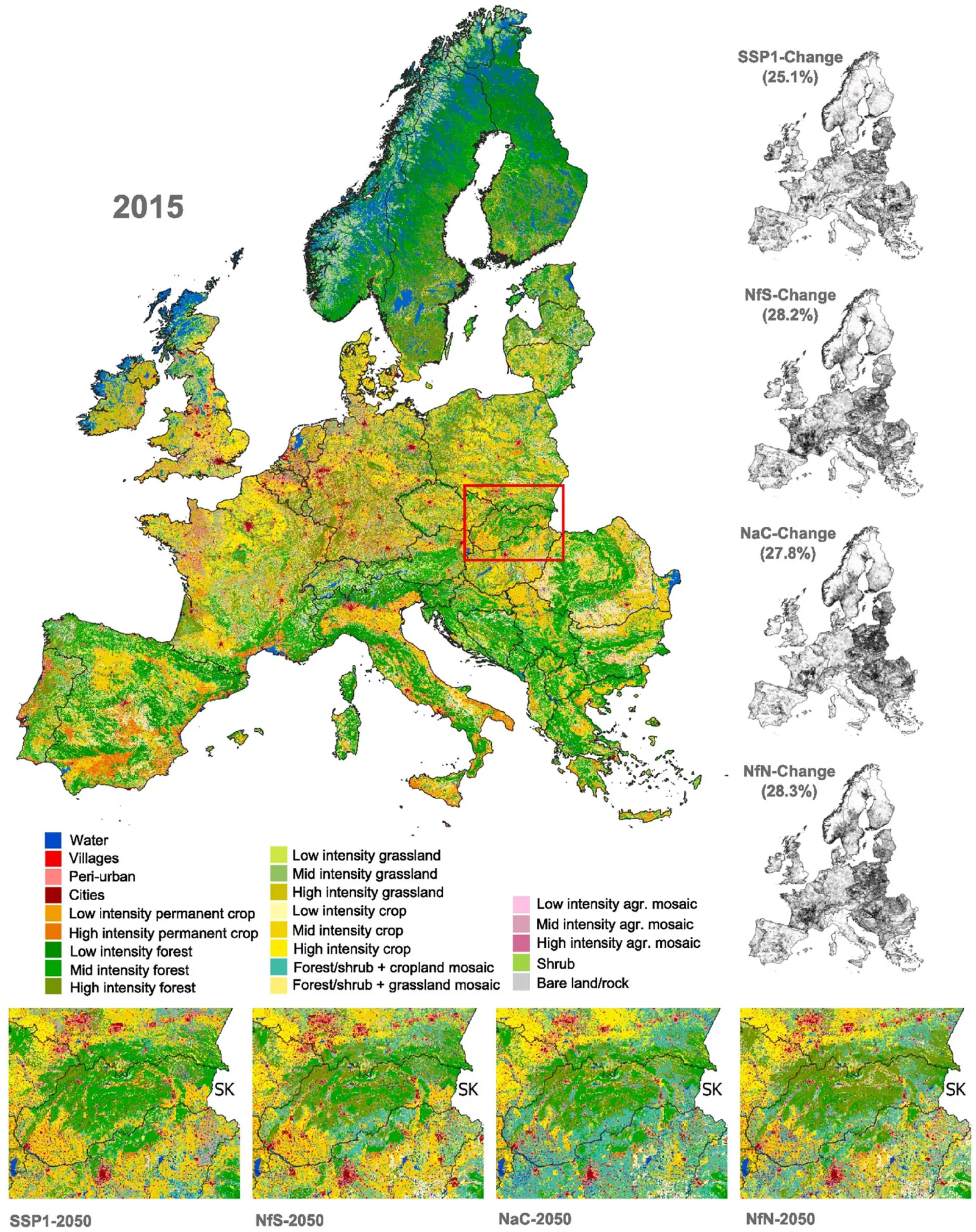
Areas undergoing land system changes between 2015 and 2050 according to the reference SSP1 and three NFF scenarios. Percent values refer to the share of European land area undergoing change in each scenario. Source: Dou et al. (2023)

Box 3: Discussing the NFF—Example of application discussing the NFF in relation to new materialist thinking to facilitate harmonious river–human relationships (Greenway 2022) This paper tackles the question of what relationships humans can develop with rivers in the twenty-first century and discusses the NFF as an example of a framework that can support the building of harmonious coexistent river–human relationships by accommodating new materialist thinking. The paper proposes a re-conceptualization of the three value perspectives of the NFF into ‘nature within society (and) society within nature’ which assumes that society encompasses culture, and that culture is not separate from society. This allows a de-centering of humans as the core influencing agent in river–human relationships and places rivers as equal actors with human actors in a holistic and interconnected dynamic between landscape, environment, and community. Acknowledging this embedded, entangled, and interconnected ontology provides a basis for conceptualizing pathways toward building harmonious river–human relationships and interactions. The paper argues that the NFF, in this context, can facilitate the development of more harmonious coexistence relationships leading to less destructive futures for both river systems and humans

Box 4: Classification tool—Example of application of the NFF in analyzing values represented in urban masterplans (Shaikh and Hamel 2023) This study aimed to understand how nature is incorporated into existing plans for new cities, and what value perspectives are represented within these plans by using the Urban Nature Futures Framework (the NFF tailored to the urban context) to conduct a content analysis on the masterplans of the Jakarta Metropolitan Area. The authors grouped the specific initiatives and actions mentioned in the masterplans into thematic categories, each coded into one or more of the NFF value perspectives based on the downstream impact of initiatives and their contribution to each value perspective. The results highlighted a predominance of the nature for society perspective aligned with the international discourse promoting nature-based solutions in urban planning. The nature for nature perspective was also prominent, but mainly in the form of reduction of environmental impact, and the nature as culture perspective, although less prominent, featured in all masterplans, suggesting a wide recognition of relational values despite the intangible nature of this perspective

**Figure:**
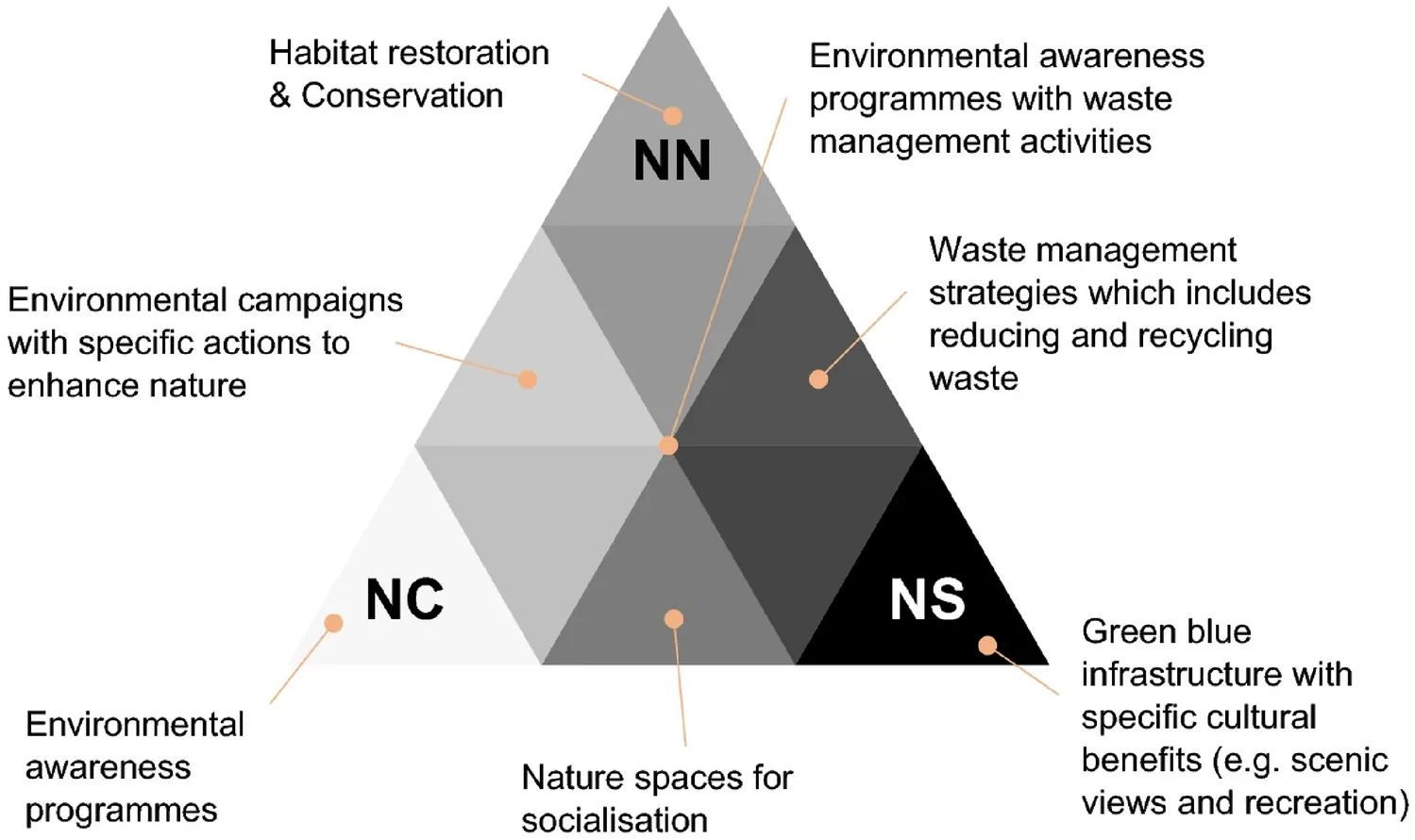
Various initiatives that are associated with different nature perspectives and their combinations. Source: Shaikh and Hamel (2023)

Box 5: Translating scenarios—Example of application of the NFF in assessing the performance of Shared Socioecological Pathways (SSPs) across normative goals (Alexander et al. 2023) This study attempted to map onto the NFF value perspectives the results from alternative SSP scenarios, each paired with an RCP consistent with the SSP storyline. The mapping was done by identifying proxy indicators for each NFF value perspective (that could be quantified with the available models and data), defining a target value range per indicator, assessing indicator outcomes for each SSP, calculating indicator scores against targeted values, and then aggregating these scores per value perspective. These calculations allowed an assessment of the performance of the different SSPs across normative goals identified for the NFF value perspectives and to compare them to 2010 baseline scores. The results showed that baseline scores already fell short of the normative goals, as did the scores for all SSPs except SSP1. Only SSP1 showed a slight increase in scores for nature for society and nature as culture, although with a decline in the score for nature for nature

**Figure:**
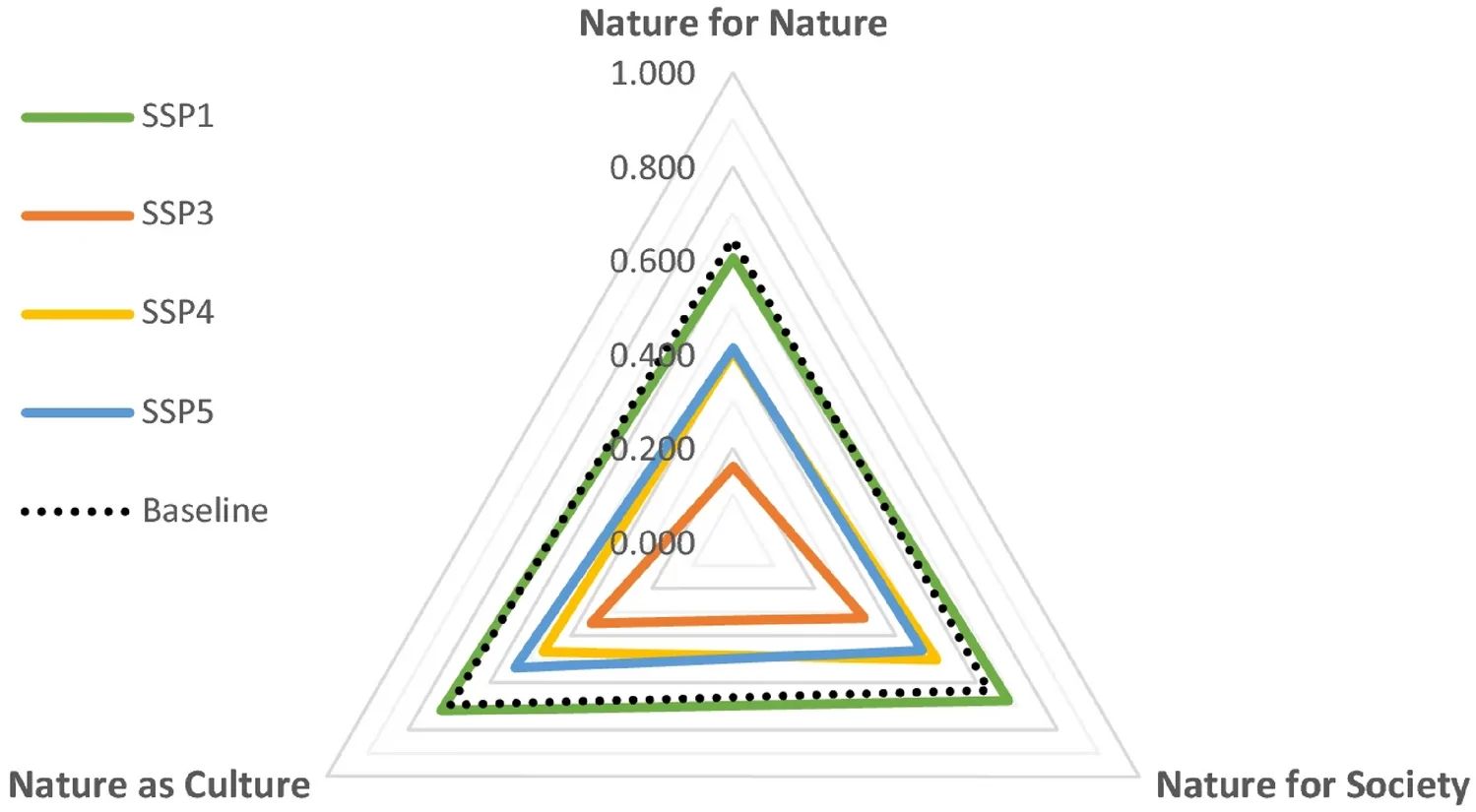
NFF value perspectives scoring plotted as a radar plot with the outcomes from SSP scenarios and the 2010 baseline. Source: Alexander et al. (2023)

Box 6: Adapting and developing models—Example of the use of the NFF to model nature-positive policy strategies and their performance across value perspectives (Haga et al. 2023) In this study, the authors developed and tested a protocol for applying the NFF to building scenarios and modeling them through a landscape-scale case study in Japan. They imposed three specifications to this exercise to align with the conceptualisation of the NFF: (1) exploring nature‑positive futures, (2) seeking alternative pathways for targets satisfying visions of plural values, and (3) screening key direct drivers to achieve the targets. The authors then applied this protocol using the LANDIS-II model to simulate land use change and vegetation succession under different forest and pastureland management scenarios under climate change. The application required a detailed zoning of the study area according to types of land use and combining these with plausible management and conservation strategies. The NFF was translated into visions and values and mapped to indicators that can be quantified by the LANDIS-II model. Using the calculated time series data of these NFF indicators, the study explored which nature-positive strategies were considered pareto optimal across NFF value perspectives and how these shifted within the NFF state space over time

**Figure:**
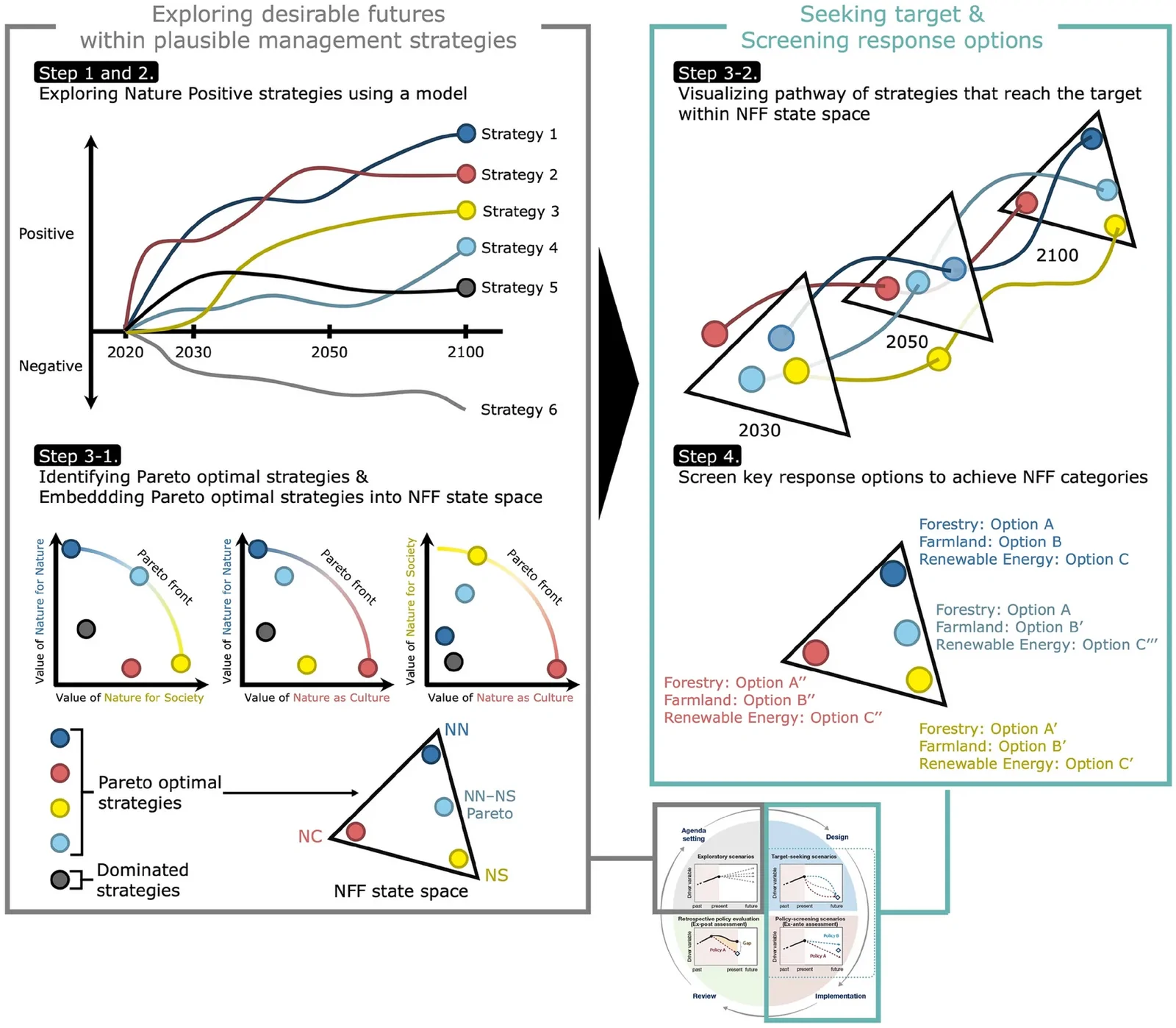
Overall protocol developed by this study, including how each step relates to the different types of scenarios and major phases of the policy cycle (bottom figure). Source: Haga et al. (2023)

Box 7: Exploration tool—Example of use of the NFF to explore the future impacts of management options under climate change (Palacios-Abrantes et al. 2022) This study used the NFF to explore multiple management options toward more desirable futures for nature under climate change. Focusing on three case studies at different ecological levels in the global South, the authors invited experts from different disciplines and backgrounds to identify how climate change impacts each value perspective of the NFF if current management practices and trends were to continue. By distinguishing positive and negative impacts on the NFF value perspectives across the case studies, the authors were able to compare the different case studies while also identifying trade-offs and co-benefits between and across NFF values. Through this study, the authors recognized the usefulness of the NFF for opening up diverse options and management pathways based on different perspectives on the values of nature that are emphasized

**Figure:**
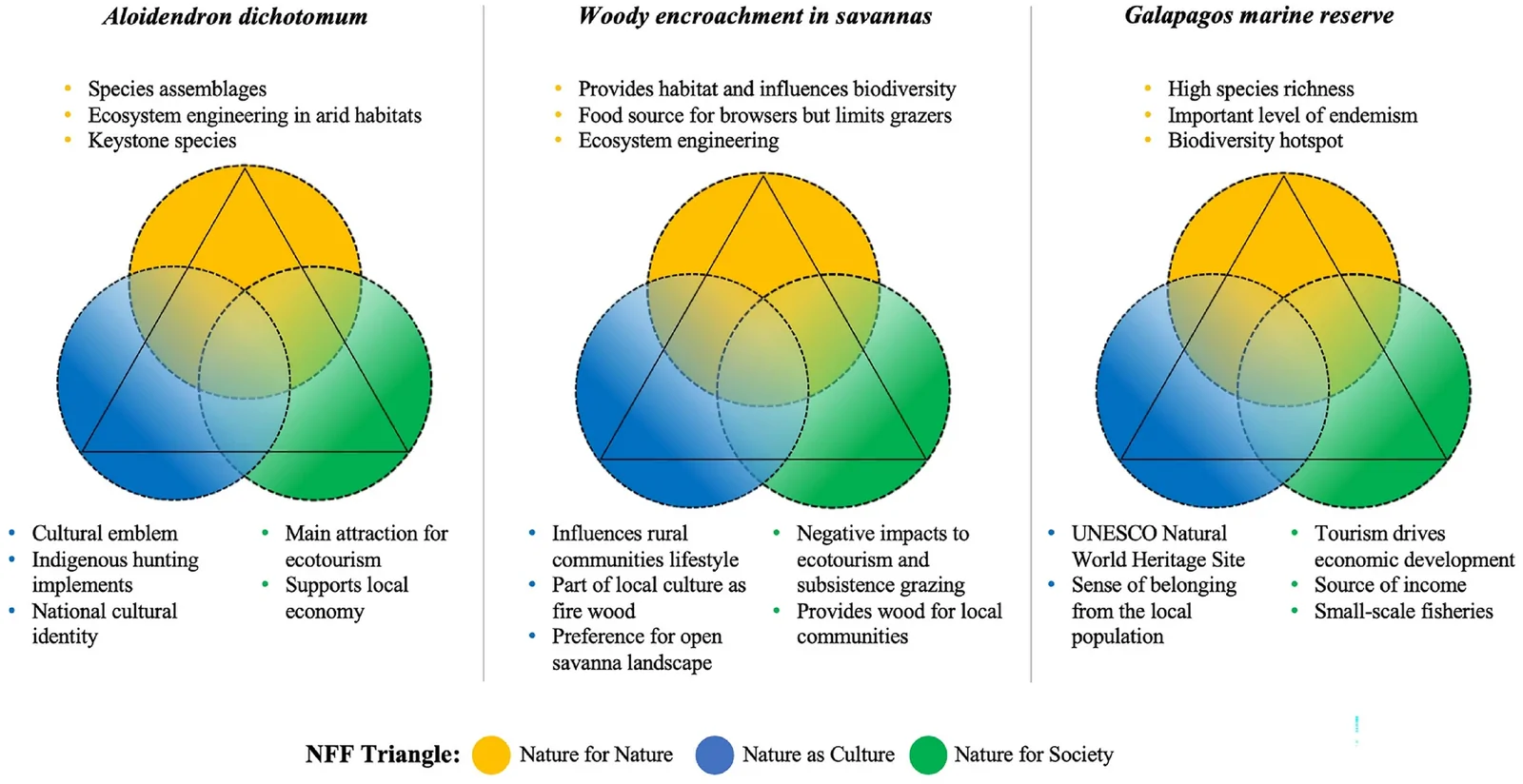
The NFF illustrating the three main value perspectives for each case study. Source: Palacios-Abrantes et al. (2022).

#### Geographic application and scale

**Fig. 2:**
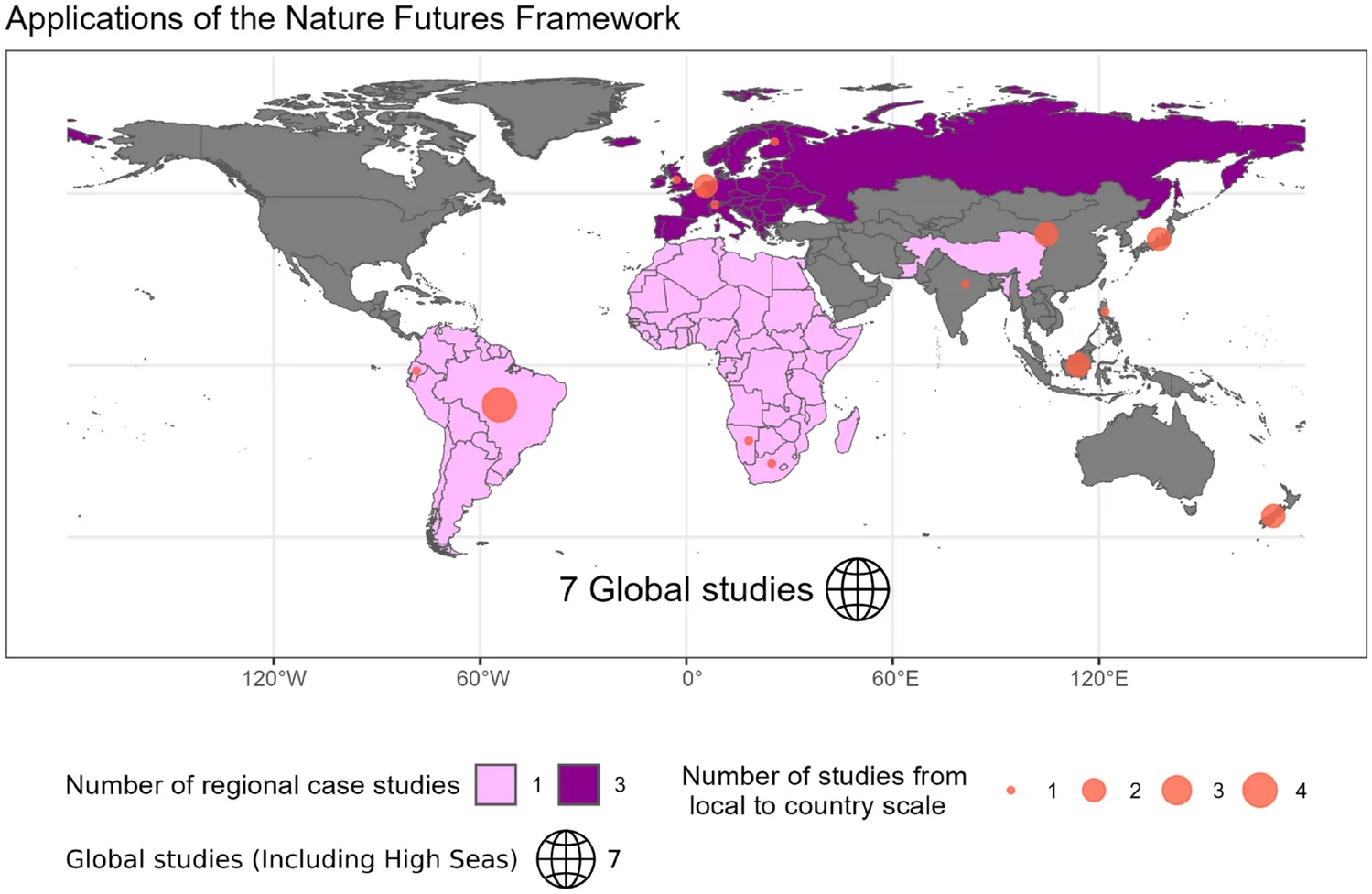
The geographical distribution of NFF applications at different spatial scales. When a single study covered multiple countries or regions, each country/region was counted, making the total number of counts (*n* = 35) higher than the total number of studies (31 studies). Aside from these 35 locations represented in the map, four studies elaborated on the concept of the NFF, with no specific geographical location

**Fig. 3:**
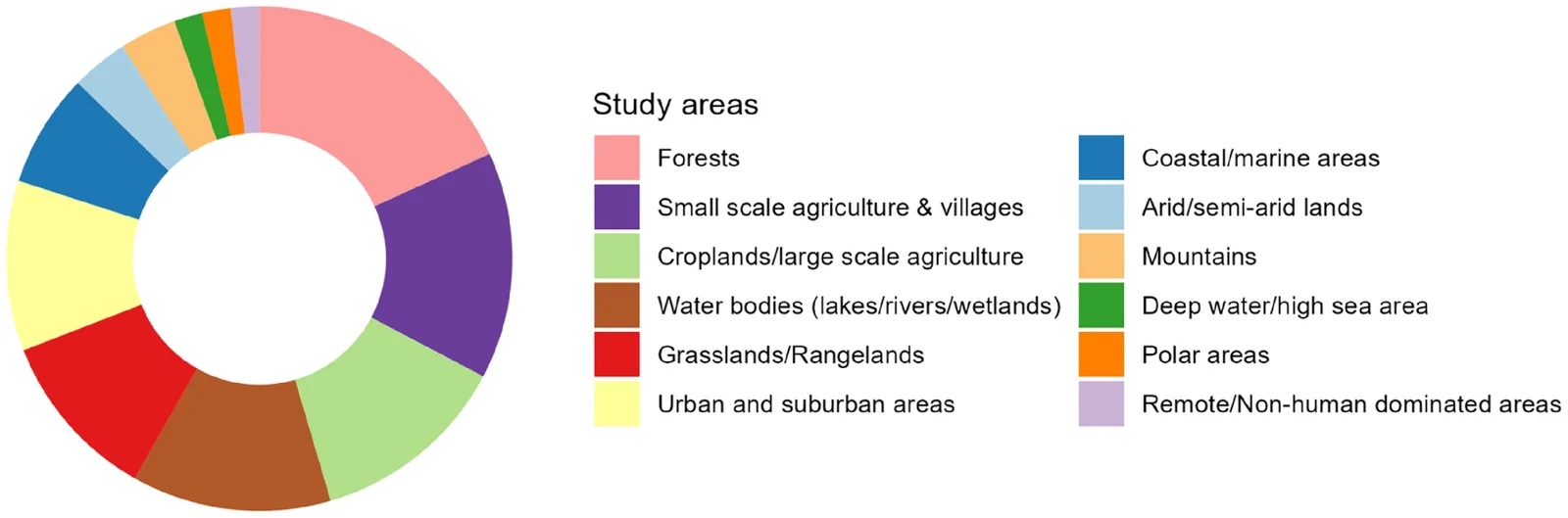
The main study areas where NFF studies have been applied. Some NFF applications cover several study areas, making the total count (*n* = 55) higher than the number of studies (31 studies)

The studies that used the NFF have a wide geographic distribution, with applications in Africa, Asia Pacific, Latin America and the Caribbean, Europe, and international waters (Fig. 2; Table S1). Applications covered all categories of spatial scales, from small landscapes to the global level (Fig. 2; Table S1). The applications covered all categories of ecosystems, including remote/non-human dominated areas through an application for the high seas. The most dominant study system was forests (Fig. 3; Table S1).

#### Methodological approaches, outputs, and relation to the policy cycle

**Fig. 4:**
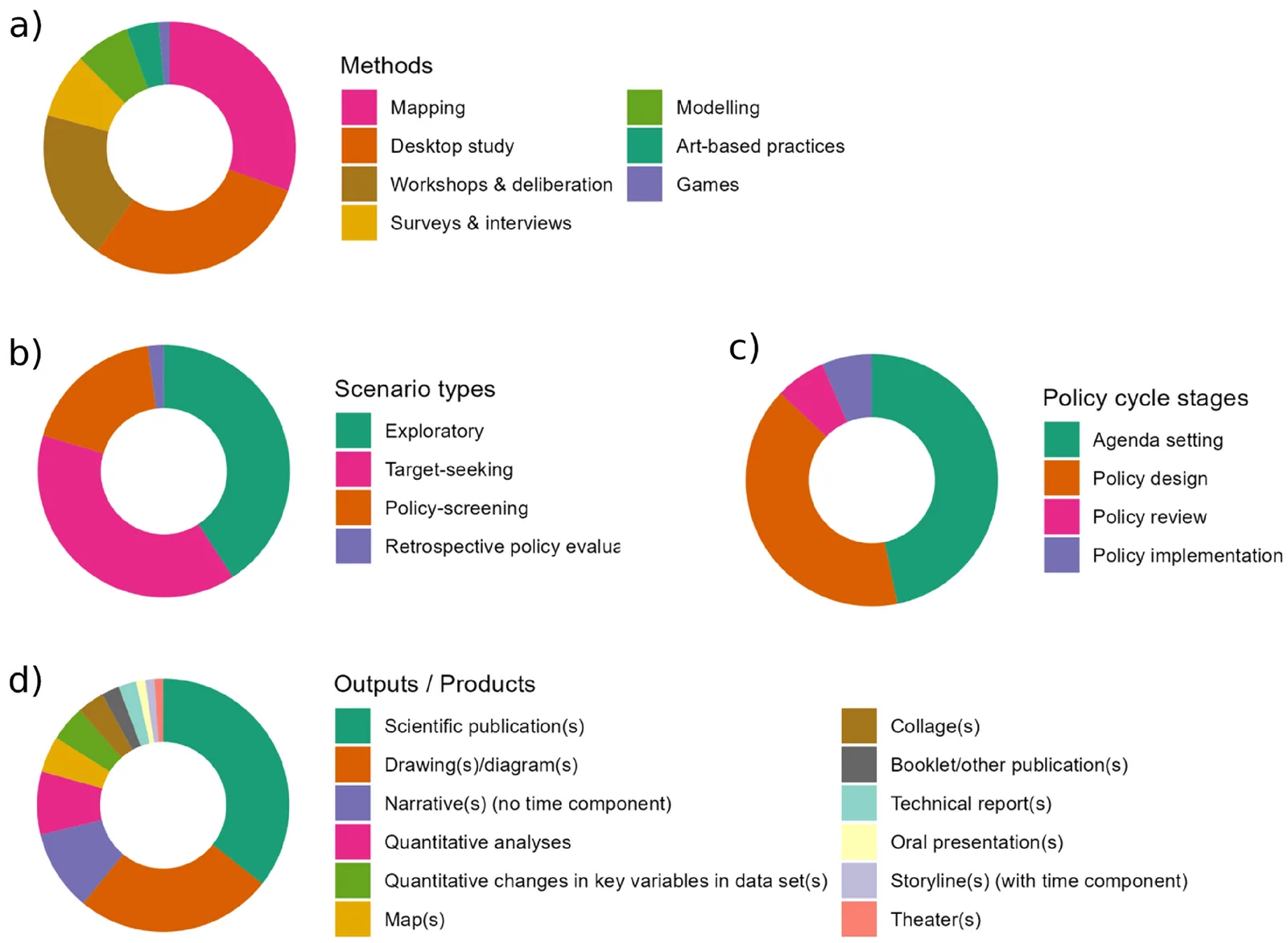
The proportions of the different: (**a**) methods (72 total counts), (**b**) scenario types (49 total counts), (**c**) policy cycle stages (62 total counts), and (**d**) outputs employed in the applications of the NFF (87 total counts). Since each study can fall into multiple categories, the total count exceeds the number of studies (31 studies)

The different applications of the NFF employed a variety of methodological approaches (Fig. 4a; Table S1). All coded categories of methodological approaches were represented, with "Desktop study", “Mapping”, and "Workshops and deliberation" being the most frequent. Many studies combined multiple approaches. Using the scenario typology employed by the IPBES methodological assessment on scenarios and models, we found that all types of scenario approaches are represented by the studies that we reviewed (Fig. 4b; Table S1), with often studies mixing approaches as they sought to explore different desirable futures by using the value perspectives as underpinning drivers of change. These diverse applications have addressed all stages of the policy cycle as employed by IPBES, with “agenda setting” as the most frequent objective (Fig. 4c; Table S1). Through these diverse methods and approaches, the studies produced a wide array of outputs, often yielding more than one type of output per study (Fig. 4d; Table S1).

#### Stakeholder participation

More than half of the studies involved some form of stakeholder input (17 of 31; Table S1). Of those, 14 involved stakeholders beyond members of the research community. Other stakeholders groups included youth, Indigenous groups, civil society organizations, natural resource managers, educators, business representatives, and policymakers, artists, researchers, and farmers/foresters/fisherfolk. Participatory efforts ranged from engagement with stakeholders in the development of NFF-based scenarios through participatory workshops or courses (e.g., Kuiper et al. 2022) to the use of NFF to interpret the results of surveys or interviews with stakeholders (e.g., Diprose et al. 2022) and involved anywhere from eight to more than 100 individuals. Ten papers include at least three different stakeholder types, giving evidence that the NFF brings together different perspectives.

#### Value perspectives and their plurality

Almost all (29 of 31; Table S1) studies addressed all three value perspectives of the NFF. One article only referred to the *Nature for Nature* value perspective, by categorizing two scenarios that were developed as *Nature for Nature* scenarios (Henry et al. 2022). There was one study that discussed the NFF, but did not explicitly engage with its value perspectives (Kluiving and Waterman 2023).

#### Global biodiversity framework and the 2030 agenda

Half of the studies mentioned the CBD’s GBF, although often this was limited to referencing the GBF in their Introduction and Discussion sections (Table S1). More substantial engagements included developing diverse illustrations of how the GBF’s vision of ‘Living in harmony with nature’ could manifest (Durán et al. 2023); presenting GBF targets and indicators to demonstrate how scenarios within different framework contexts may be expressed (Kim et al. 2023) and using specific targets as inputs for quantitative scenario development to show various ways these targets can be achieved (Dou et al. 2023). Similarly, half of the studies mentioned the Agenda 2030’s SDGs (Table S1), but also mostly in the Introduction and Discussion section, and only few listed specific SDGs. An example of a study that engaged more thoroughly with the SDGs is Kuiper et al. (2022) which conducted an SDG target analysis of the co-produced visions of a national park to understand how achieving these visions would contribute to sustainable development. Besides the GBF and the SDGs, studies on the NFF were positioned in relation to various other international bodies and agreements, including the IPCC (Palacios-Abrantes et al. 2022), Ramsar Convention on Wetlands (Sarkar et al. 2020), and the UN Decade on Restoration (Dunn-Capper et al. 2023).

#### Nature’s contributions to people and transformative change

In 18 of the 31 studies, the concept of NCPs is mentioned (Table S1). However, in most of these cases, it is used only to point to the benefits of nature for people in a general sense, without applying the NCP classification to analyze specific NCPs. A notable example of a study that engaged more deeply with the concept of NCP is from Quintero‐Uribe et al. (2022), which used NCPs alongside the NFF to analyze existing participatory scenarios and quantify changes in a variety of NCPs.

The majority of studies (25 of 31) positioned themselves in the context of transformative change by mentioning the concept (Table S1), although this includes studies that mentioned the need for transformation only in the Introduction and Discussion sections without further operationalization. Studies that engaged more deeply with the concept of transformation highlight the importance of engaging peoples’ plural values and the development and analysis of visions and pathways to navigate transformation, using—besides the NFF—frameworks such as the Three Horizons (e.g., (Kuiper et al. 2022)), Seeds of Good Anthropocenes (e.g., (Mayer et al. 2023), Leverage points (e.g., (Rana et al. 2020) and the Iceberg model (Pereira et al. 2023).

#### Limitations and feedback on the NFF

The studies included in this review highlighted some strengths and limitations of the NFF with suggestions for further development and consideration (Table S1). The integrative, holistic, inclusive, and open feature of the framework was highly appreciated (Henry et al. 2022; Diprose et al. 2022; Palacios-Abrantes et al. 2022; Stronge et al. 2023) and the framework was perceived to allow new ways of thinking and developing positive and environmentally conscious future pathways with transparency and flexibility (Resende et al. 2020; Greenway 2022; Quintero‐Uribe et al. 2022; Lahoti et al. 2023; Dunn-Capper et al. 2023). The majority of limitations and challenges were on conflicting views on nature value perspectives (Greenway 2022; Stronge et al. 2023; Alexander et al. 2023), challenges with different normative assumptions and subjectivity (Rana et al. 2020; Haga et al. 2023; Mayer et al. 2023), limited capability and tools around cultural/relational values (Sarkki et al. 2023; Kramer et al. 2023), complexity in analyzing or resolving tensions and trade-offs (Pereira et al. 2020; Rana et al. 2020), and resource intensiveness (Palacios-Abrantes et al. 2022; Sarkki et al. 2023). Future work is suggested to further understand normative assumptions underlying value perspectives and transformation (Durán et al. 2023; Dou et al. 2023), and for quantification and testing of indicators (Haga et al. 2023; Kramer et al. 2023; Kim et al. 2023; Shaikh and Hamel 2023). The importance of relativity across value perspectives rather than extremes or as a standalone (Sarkki et al. 2023; Durán et al. 2023; Mayer et al. 2023) and working with existing tools and frameworks in optimizing the uptake of the NFF were also recommended (Kim et al. 2023; Shaikh and Hamel 2023).

## Discussion

In response to key gaps identified by the 2016 IPBES methodological assessment on scenarios and models, the NFF was introduced to catalyze the development of new scenarios and models that more effectively address IPBES's needs. In this discussion, we evaluate the findings of our literature review concerning the key areas where the NFF aims to drive progress—referred to as the "promises". For each promise, we begin with briefly recapping the intended ambition, then assess the extent to which this ambition is being realized, and conclude by highlighting the remaining development needs. Subsequently, we reflect on limitations of our study, including the focus on scientific literature, recognizing that scenario and model outputs are often presented in the gray literature and in languages other than English. Finally, we reflect on the implications of the uptake of the NFF in the context of IPBES and CBD.

### On the promise of catalyzing scenarios and models of positive nature futures

The IPBES Methodological Assessment on Scenarios and Models (2016) found that most scenario studies in the literature do not prioritize nature and biodiversity in their narratives, and when nature and biodiversity are considered, scenarios typically reveal a continued decline (Rosa et al. 2017, 2020b). An important objective of the NFF is to drive the exploration of futures in which the web of life is restored (Pereira et al. 2020).

Our review reveals that applying the NFF ensures a specific focus on nature and biodiversity in all studies. Importantly, it indicates that it at least seems feasible to *imagine* positive futures, as a dozen studies presented positive visions of the future for nature and people (Table 1). Some of these studies also explored policy options and other measures for achieving the envisioned futures (Box 6). While most scenario descriptions remain qualitative, Dou et al. (2023) highlight the potential for integrated quantitative analysis of positive futures using the NFF, building on the SSP1 “Taking the Green Road” scenario (Box 2).

To inform upcoming IPBES assessment reports, there is a strong need for more integrated, quantitative studies that address multiple spatial scales and policy objectives. These studies could provide valuable insights into how existing scenarios and modeling frameworks can be effectively integrated into environmental assessments, while also highlighting areas that require further development. An important step in this regard is the work of Alexander et al. (2023), who mapped the SSP scenarios (initially developed for the IPCC) onto the NFF (developed for IPBES), establishing a vital link between the two frameworks (see Box 5). Another research priority involves conducting follow-up assessments to gauge the tangible impact of scenario studies employing the NFF, as the ultimate success of the NFF can be evaluated through observable improvements for people and nature in the biosphere.

### On the promise of catalyzing scenarios that engage with plural values

How societies value nature and biodiversity, and how values are considered in decision-making, determine which future we are heading toward. The IPBES Values Assessment (IPBES 2022) showed that the way nature is valued represents both a key driver of biodiversity loss and a potential leverage point for transformative change.

Our review showed that almost all studies did engage with all three value perspectives of the NFF, demonstrating its effectiveness as a tool for considering multiple values of nature. Palacios-Abrantes et al. (2022) highlighted the NFF's capacity to offer insights into the trade-offs and synergies that may occur within and between different value perspectives, allowing policymakers to make better informed decisions. However, they also stated that such insight into trade-offs does not necessarily facilitate decision-making, as indicators on each side of the trade-off or synergy can be difficult to measure and implement. Particularly, challenges arise in assigning indicators to the relational "nature as culture/one with nature" perspective, with participants sometimes struggling to differentiate it from nature for society (Rosa et al. 2020a; Haga et al. 2023). Additionally, while the nature as culture/one with nature perspective aims to be inclusive of non-Western viewpoints, Stronge et al. (2023) caution that a science-based framework may inherently carry a Western-centric bias.

Most studies have concentrated on the three value perspectives located at the corners of the NFF triangle, but more research is needed to operationalize the interior space of the framework. Approaches to this can differ depending on the study's objectives. For example, a deductive, quantitative approach might begin by defining the corner perspectives and then position scenarios within the triangle relative to these points. Alternatively, an inductive, qualitative approach could start by analyzing a specific location within the triangle, describing its distinct features and indicators before making comparisons with other areas. Additionally, further analysis is required to explain the normative assumptions underlying the positions within the triangle and their broader, political implications.

### On the promise of catalyzing scenarios that are multi-scale

As global human–environment interactions become increasingly complex, with feedbacks spanning diverse domains and drivers, an integrative approach to future planning is crucial. Both the Millennium Ecosystem Assessment (2005) and the IPBES Methodological Assessment on Scenarios and Models (2016) emphasized the need for scenarios that move beyond single-scale perspectives to capture the dynamic interplay between human and natural systems. Although policies are typically implemented at local levels, they are shaped by broader regional frameworks, making it essential for scenarios to address cross-scale interactions while remaining context specific and grounded in local realities.

Our review demonstrates that the NFF is applicable in a diversity of systems at any regional scale. Diverse future visions and pathways have been explored with stakeholders or through knowledge generation and synthesis at the local or landscape level (Quintero-Uribe et al. 2022; Kuiper et al. 2022; Dunn-Capper et al. 2023; Haga et al. 2023), in different ecosystems including the river basin, forest, mountains and wetlands (Lembi et al. 2020; Sarkar et al. 2020; Dasgupta and Shakya 2023), or within administrative boundaries at city (Shaikh and Hamel 2023), provincial (Pan et al. 2022), national (Yu et al. 2021), continental (Dou et al. 2023), and global (Alexander et al. 2023; Durán et al. 2023; Pereira et al. 2023) levels.

However, a significant gap remains in current applications: no studies have yet integrated scenarios and models across multiple scales within a single project. Although resource-intensive, conducting comparative or complementary scenario analyses could provide valuable insights into how to synergize and address scale-related challenges. For example, in climate and biodiversity interactions, both spatial and temporal scales must be considered, as some impacts are observable in the short term, but may have compounding and cascading effects over the long term, from local ecosystems to the global atmosphere (Pörtner et al. 2021).

### On the promise of catalyzing scenarios of transformative change

Various integrated assessment studies on biodiversity and ecosystem services have highlighted the necessity for transformative change to achieve sustainability targets, yet much remains unclear about the specifics of such transformation and how it can be initiated and navigated. Within IPBES, this has led to an assessment dedicated to transformative change and, in the context of scenarios and models, to the development of the NFF as a tool to catalyze and describe transformative change.

The review shows that the NFF has been particularly effective in visioning studies, providing a foundation for creating positive futures for nature and people. These studies inherently envision transformed futures, acting as attractors that can initiate and guide change, while identifying and inspiring the necessary transformative actions. However, more research is needed to explicitly engage with theoretical or conceptual frameworks of transformative change. Current studies often reference these frameworks heuristically, without fully developing transformative pathways or quantifying impacts.

Future studies could focus on systematically addressing the anthropogenic drivers causing current unsustainable practices, such as climate change, chemical pollution, resource overexploitation, and invasive species. There is also a clear research need for quantitative studies that directly engage with the targets and indicators of the SDGs as well as the Kunming–Montreal GBF. Such studies are essential to assess the extent to which transformative visions and pathways can deliver biosphere-based sustainability.

### On the promise of catalyzing scenarios that are co-produced

Dominant global-scale scenario development approaches frequently exclude diverse values and knowledge systems, thereby reducing the relevance and potential positive impact of these scenarios (Rosa et al. 2017; Beck and Forsyth 2020; Pereira et al. 2021a). In contrast, the NFF was designed to incorporate diverse value perspectives and knowledge systems (including local and traditional ecological knowledge) via participatory scenario development (Pereira et al. 2020).

Over half of the papers in our sample included some stakeholder involvement, and those that did exhibited considerable diversity in the type, number, and ways in which stakeholders were engaged. Of the studies that focused specifically on developing visions and scenarios, the majority engaged stakeholders, either through participatory processes of visioning or through workshop deliberations. This subset of studies also presented the largest variety of tools and techniques for stakeholder participation which could be combined with the NFF (Table S1). These observations indicate that the NFF is capable of enabling a shift toward more inclusive and pluralistic approaches to scenario development. Indeed, several of these studies reflected on the capability of the NFF to transparently capture different value perspectives and support stakeholder-driven scenario development while maintaining consistency and comparability across such diverse scenarios. On the other hand, all quantitative studies using the NFF remained expert based (Table 1), highlighting the challenge of bridging the creative process of participatory visioning with existing quantitative indicators and models.

Building out a broader set of NFF applications that engage stakeholders in scenario development would increase the diversity of scenarios and enable more comprehensive comparison and identification of best practices. To that end, it would be useful for future NFF studies to include an evaluation component that more formally tracks the effectiveness of different means of engaging stakeholders in NFF scenario development.

### On the promise of being a flexible tool

The NFF was designed from its inception to be adaptable across various contexts, based on user needs. It was further developed as a tool primarily focused on facilitating the creation of scenarios that explore diverse and transformative perspectives on desirable futures for both nature and people (Pereira et al. 2020). When presented at the 9th session of the IPBES Plenary in 2022, member state negotiations emphasized the importance of the NFF's flexibility to avoid it being perceived as prescriptive. This flexibility is central to the methodological guidance for the NFF (IPBES 2023).

The results of this review confirm the success of this flexible approach, with the NFF being applied across all six use categories. While it is frequently used for developing visions and scenarios for nature in line with its original purpose, its utility extends beyond participatory scenario development. The NFF has been employed to elicit and categorize diverse values people hold about nature, as well as to identify where further methodological developments are needed in indicators and models. Its versatility is further evident in its application across the stages of the policy cycle, from agenda setting and policy design to implementation and review.

The complexity of place-based social–ecological systems demands a variety of approaches and tools to capture the diverse values people hold and the futures they envision. This review highlights that the research community is increasingly responding to calls for more inductive, bottom-up scenarios, as recommended in past IPBES assessments. In this context, the NFF is driving a paradigm shift toward a values-based exploration of positive futures across multiple disciplines, rather than adhering to a prescriptive approach. However, questions remain as to whether the NFF can fully deliver on its ambitions, particularly in identifying concrete broadly supported pathways to achieve the envisioned futures or in evaluating their long-term sustainability. The relatively low number of quantitative applications of the NFF suggests that further testing, assessment, and refinement are necessary to establish best, resource efficient practices.

### Limitations of our study

Reviewing scientific literature through Scopus has several advantages, including a focused examination of an important target group for uptake of the NFF—the scientific community. It promotes transparency and reproducibility of the search outcomes and leverages the fact that all sources are peer reviewed. However, this approach comes with inherent limitations. It neglects articles in other languages as well as articles residing in the gray literature. This is problematic since a substantial body of work on scenarios and modeling, including reports on the NFF, may be in the gray literature.

A semi-structured review of NFF literature prepared by the Technical Support Unit of the Task Force on scenarios and models for the 10th plenary session of IPBES in 2023 (IPBES/10/INF/13) encompassed work not covered by our review, such as a book chapter, more popular science articles, workshop reports, a pre-print, a doctoral thesis, and several MSc thesis reports (see https://www.zotero.org/groups/4937409/nature_futures_framework/items/97JRX9TV/library). This also included several peer-reviewed articles on the NFF that were not picked up by our Scopus search, either because the NFF was not mentioned in the title, abstract, or keywords (e.g., Siqueira-Gay et al. 2020) or because the work was published after our search (e.g., Otero et al. 2024). To fully grasp how the NFF achieves its objectives, future literature reviews and assessments could contemplate embracing a more comprehensive strategy to include gray literature and sources in diverse languages that does not compromise reproducibility.

To provide an initial overview of the evolving literature around the NFF, descriptive statistics were sufficient for this study. However, with a larger dataset, more sophisticated analyses such as cluster analysis could be employed to uncover deeper insights into patterns and trends, potentially revealing distinct thematic clusters that offer valuable perspectives on the NFF’s development and application. Further research could also delve deeper into the current categories, using content analysis to clarify which specific SDGs or NCPs the studies address, for example. Additionally, maintaining a database of indicators, associated models, and relevant data for quantification would be valuable for catalyzing future studies.

Our reliance on peer-reviewed literature tends to be retrospective, primarily offering insights into completed work. A more forward-looking analysis of ongoing NFF applications would offer additional evidence of uptake and complementary insights into best practices. To provide a sense of work that is underway, we present an overview of ongoing or recently completed large international research projects employing the NFF (See Table S2). The information is gathered from the articles in our dataset, IPBES workshop reports, and the IPBES Impact Tracking Database (TRACK). This collection of ongoing research projects underscores a notable interest from major funding bodies in supporting research in this domain, albeit with a dominance by European initiatives. Overlapping with many of the institutions undertaking these projects is a community of practice on Biodiversity and Ecosystem Services Scenarios-based Model Intercomparison (BES-SIM), consisting of integrated assessment modelers and biodiversity and ecosystem services modelers.

### Implications of NFF uptake in the context of IPBES and CBD

The NFF complements conventional approaches to developing scenarios focusing on the human impacts on nature and nature’s contributions to people under future uncertainties, by allowing the description of desirable futures and the exploration of pathways to reach such futures. The NFF also helps people recognize and articulate multiple values of nature and nature’s contributions to people. As illustrated by the diverse uses identified in this paper, the NFF may inform ongoing and future IPBES assessments by not only generating new visions and scenarios, but also serving as a framework to organize and assess existing knowledge and policies from a plural values perspective, and to highlight knowledge gaps or omissions in values considerations. Furthermore, IPBES has mandated its task force on scenarios and models to promote dialogue between IPBES and the communities of practice on scenarios and models. With the increasing uptake of the NFF in recent years, as evidenced by the expansion of authors of NFF studies beyond those of the initial publication of Pereira et al. (2020), a community of scientists and stakeholders is growing around this tool. This community would be able to provide valuable knowledge to IPBES’s future work, including assessments, capacity building, and policy support. Ideally, this mandate of the task force on scenarios and models serves as momentum to bring the stakeholders engaged in past or ongoing applications of the NFF together as a community of practice—an initiative under development by the current and former members of the task force (https://www.naturefuturesframework.org/).

In the context of the Convention on Biological Diversity, the plural values of nature have gained increasing recognition since the Strategic Plan for Biodiversity 2011–2020 was formulated, accelerated by the launch of the IPBES Values Assessment in 2022. Currently, target 14 (Integrate Biodiversity in Decision-Making at Every Level) of the Kunming–Montreal Global Biodiversity Framework explicitly recognizes the multiple values of nature and requires its full integration into policy instruments alongside consideration for biodiversity. The NFF would constitute a versatile tool to support this target, and due to its applicability in participatory processes, may also contribute to other targets such as target 22 (Ensure Participation in Decision-Making) or 23 (Ensure Gender Equality and a Gender-Responsive Approach) depending on its usage.

## Conclusions

In spite of its infancy, the NFF has already been used for a wide variety of applications in many different countries and socioecological contexts. Since the initial publication of the NFF in 2020, there has been a continuous uptake of the framework. Numerous visioning and scenario development exercises have been conducted using the NFF, and it has been extensively discussed in the scientific literature. Despite some identified limitations and remaining challenges, the NFF is valued for allowing the consideration of diverse perspectives on nature and developing scenarios that reflect pluralistic values. This contributes to various developments and knowledge needs as previously established by the IPBES assessment and other research agendas. Given the Anthropocene challenges we face and the potential of scenario analysis to address them, this is an important first step, but the rollout of the NFF is still work in progress, and we identified several areas where future research with the NFF is still needed. This includes the integration of the framework across multiple scales; enhancement of quantitative methodologies; further operationalization of transformative change; and addressal of the complexities of incorporating diverse cultural and relational values. Ongoing collaboration with communities of practice and further research is crucial to enhance the framework's effectiveness. We hope that this article conveys the potential of the NFF to the broader research community and will contribute to achieving the CBD's vision of living in harmony with nature.

## Supplementary Information

Below is the link to the electronic supplementary material.

Supplementary file1 (XLSX 471 KB)

Supplementary file2 (DOCX 30 KB)

## Data Availability

All data supporting the findings of this study are available within the paper and its Supplementary Information. Detailed information on the diverse uses of the NFF extracted from the reviewed studies, as well as the variables and codes, can be found in Table S1.
