# Supplementary material for: Catalyzing change: a literature review on the implementation of the Nature Futures Framework
Source: Sustain Sci. 2025 Jun 27;21(5):2277–96. doi: 10.1007/s11625-025-01682-y (PMC13680305; doi:10.1007/s11625-025-01682-y)
Supplement: Supplementary file 1 [file 11625_2025_1682_MOESM2_ESM.docx]

| Table S2 A non-exhaustive overview of research projects using the NFF | | | |
| --- | --- | --- | --- |
| Project | Funder | Description | Website |
| NaturaConnect | EU Horizon Europe | NaturaConnect aims to build a coherent and resilient Trans-European Nature Network (TEN-N) of conserved areas across Europe for nature and people. The NFF is used to develop quantitative nature futures scenarios. | https://naturaconnect.eu/ |
| BioNext | EU Horizon Europe | The project produces new evidence to better understand biodiversity loss and demonstrates how biodiversity underpins every aspect of life. The NFF is used for exploring new visions for nature-centered Europe for 2050. | https://www.bionext-project.eu/ |
| wildE (Making rewilding climate smart) | EU Horizon Europe | wildE develops climate-smart rewilding as a nature-based solution to the twin threats of climate change and biodiversity loss. The NFF is considered for the development of rewilding scenarios. | https://www.wilde-project.eu/ |
| FinBIO (MISTRA Finance to Revive Biodiversity) | Swedish foundation for strategic environmental research MISTRA | FinBIO’s mission is to support the financial sector’s capacity to contribute to a nature-positive economy, enhancing the resilience of our planet by reversing the loss of nature and biodiversity. The NFF is used in developing scientifically grounded techniques to plan, design, implement, and evaluate nature positive investments. | https://finbio.org |
| DRYvER (Securing biodiversity, functional integrity and ecosystem services in DRYing riVER networks) | EU Horizon 2020 | DRYvER studies how biodiversity, ecosystem functions, ecosystem services and their values in drying river networks are directly and indirectly altered by climate change through empirical and modelling work, and develops knowledge-based strategies and tools for cost-effective adaptive management of drying river networks. The NFF was used in a participatory workshop to envision a sustainable river future. | https://www.dryver.eu/ |
| Ponderful | EU Horizon 2020 | The PONDERFUL project investigates how ponds can be used as nature-based solutions (NBS) for climate change adaptation. The NFF was used in participatory workshops to elicit stakeholder values of ponds. | https://ponderful.eu/ |
| TerraNova | EU Horizon 2020 | TerraNova is a landscape learning initiative, with a mission to develop a digital atlas of Europe that combines human population patterns in the past, plants and disturbances, animal development, and climate change. The NFF was used in several landscape restoration studies. | https://www.terranova-itn.eu/ |
| CABES (Capacity Development for Biodiversity and Ecosystem Services Experts) | German Federal Ministry for the Environment, Nature Conservation, Nuclear Safety and Consumer Protection (BMUV) | CABES aims to develop and strengthen the capacity of professionals in biodiversity-related fields in West, Central, and East Africa to engage in the Intergovernmental Science-Policy Platform on Biodiversity and Ecosystem Services. The NFF has been introduced in one of its first regional capacity-building workshops. | https://www.cabes.online/ |
| Future Ecosystems for Africa (FEFA) | Oppenheimer Generations Research and Conservation (OGRC) | FEFA supports research for conservation and development across the continent by managing a portfolio of projects to create diverse African-led futures, support cutting-edge multidisciplinary science in Africa, and empower African citizens to drive and respond to global change. The NFF has been applied in various case studies under this program, ranging from workshops and reports, to eco-fiction novels. | https://futureecosystemsafrica.org/ |
| S-21 IAM-B (Development of an Integrated Assessment Model linking Biodiversity and Socio-Economic Drivers, and its Social Application) | Japanese Environment Research and Technology Development Fund (ERTDF) | IAM-B aims to develop an integrated assessment model which can consider biodiversity, climate change, and other socio-economic drivers in an integrated manner for future scenario analysis, and quantitatively estimate the impacts of response options for climate change mitigation and adaptation, and biodiversity conservation and restoration under different future scenarios in Japan. The NFF is used to formulate future scenarios and combinations of response options. | https://iam-b.jp/en/ |
| BES-SIM2 | Grassroots funding from engaged institutions | A global consortium of scenario researchers that have initiated a comparative exercise of NFF scenarios to be completed in time to inform the 2nd IPBES Global Assessment, building on the Biodiversity and Ecosystem Services Scenarios-based Model Intercomparison (BES-SIM) that informed the 1st IPBES Global Assessment in 2019 | In prep |
| Korean Nature Futures  (Developing a globally useful visioning and forecasting framework for biodiversity, people, and climate neutrality in Korea) | National Capability for Global Challenge (UK Research and Innovation) | KNF develops ecologically diverse and harmonious future scenarios of the Korean peninsula with DMZ as global commons using the NFF and diverse innovations in informing international and national policy frameworks. It advances the co-development of narratives and modelling framework with a broad range of stakeholders via the DMZ Open Festival’s Ecopeace Forum with a regional provincial office. The project joins a suite of models for nexus systems analyses and social-ecological feedbacks modelling to inform Korea’s transformative pathways. | https://ggtour.or.kr/dmzopen  (project site under development - may be ready by the publishing date of this paper) |
